# Significant Beneficial Association of High Dietary Selenium Intake with Reduced Body Fat in the CODING Study

**DOI:** 10.3390/nu8010024

**Published:** 2016-01-04

**Authors:** Yongbo Wang, Xiang Gao, Pardis Pedram, Mariam Shahidi, Jianling Du, Yanqing Yi, Wayne Gulliver, Hongwei Zhang, Guang Sun

**Affiliations:** 1 Department of Endocrinology, the First Affiliated Hospital of Dalian Medical University, Dalian 116000, Liaoning, China; 271975039@qq.com (Y.W.); dujianling63@163.com (J.D.); 2 The Discipline of Medicine, Faculty of Medicine, Memorial University, St. John’s, NL A1B 3V6, Canada; xx526916212@126.com (X.G.); pedram.pardis@gmail.com (P.P.); drgulliver@newlabresearch.com (W.G.); hongwei8888@hotmail.com (H.Z.); 3 College of Food Science and Engineering, Ocean University of China, Qingdao 266003, Shandong, China; 4 Division of Endocrinology and Metabolism, Department of Medicine, University of Alberta, Edmonton, AB T6G 2G3, Canada; mshahidi@ualberta.ca; 5 Division of Community Health & Humanities, Faculty of Medicine, Memorial University, St. John’s, NL A1B 3V6, Canada; yanqing.yi@med.mun.ca

**Keywords:** dietary selenium intake, body composition, confounding factors, adult population

## Abstract

Selenium (Se) is a trace element which plays an important role in adipocyte hypertrophy and adipogenesis. Some studies suggest that variations in serum Se may be associated with obesity. However, there are few studies examining the relationship between dietary Se and obesity, and findings are inconsistent. We aimed to investigate the association between dietary Se intake and a panel of obesity measurements with systematic control of major confounding factors. A total of 3214 subjects participated in the study. Dietary Se intake was determined from the Willett food frequency questionnaire. Body composition was measured using dual-energy X-ray absorptiometry. Obese men and women had the lowest dietary Se intake, being 24% to 31% lower than corresponding normal weight men and women, classified by both BMI and body fat percentage. Moreover, subjects with the highest dietary Se intake had the lowest BMI, waist circumference, and trunk, android, gynoid and total body fat percentages, with a clear dose-dependent inverse relationship observed in both gender groups. Furthermore, significant negative associations discovered between dietary Se intake and obesity measurements were independent of age, total dietary calorie intake, physical activity, smoking, alcohol, medication, and menopausal status. Dietary Se intake alone may account for 9%–27% of the observed variations in body fat percentage. The findings from this study strongly suggest that high dietary Se intake is associated with a beneficial body composition profile.

## 1. Introduction

The prevalence of overweight and obesity has risen substantially in the past three decades. A recent survey showed that the worldwide prevalence of overweight and obesity increased by 27.5% for adults and 47.1% for children between 1980 and 2013, with the number of overweight and obese individuals soaring from 857 million to 2.1 billion during the same period [1]. Obesity is associated with a multitude of chronic medical conditions, including type 2 diabetes, heart disease, hypertension, stroke, and certain types of cancer [2]. In 2010, overweight and obesity were estimated to have caused 3.4 million deaths, 4% of years of life lost, and 4% of disability-adjusted life-years worldwide [3]. Given its substantial increase in prevalence and associated health risks, obesity has become a major global health challenge. It is influenced by various factors, including genetic predisposition, variations in nutrient intake, and behavioral and environmental factors [4]. The important contribution of macronutrient intake to obesity has become better recognized [5]. In addition, recent studies have suggested that certain micronutrients may also be associated with increased body fat accumulation [6,7,8].

Selenium (Se) is a nutritionally essential trace element and naturally presents in many foods. Its biological effect is exerted via incorporation into selenoproteins, which play a critical role in reproduction, thyroid hormone metabolism, DNA synthesis, and protection from oxidative stress and inflammation [9]. There is abundant evidence linking low Se status with the development of several chronic diseases, including cardiovascular disease [10,11], cancer [12,13], and diabetes [14,15,16].

Some studies have suggested that Se may also inhibit adipocyte hypertrophy and adipogenesis [17,18]. Furthermore, biomarkers of Se nutrition status including serum Se levels as well as the activity of the important selenoprotein, glutathione peroxidase (GPx), may be associated with obesity [19,20,21]. However, existing data were obtained mainly as byproducts from a few studies originally designed to study diabetes and cancer rather than obesity. In addition, the reported results were inconsistent. Obesity status in all studies was estimated by body mass index (BMI) or waist circumference (WC), which have limited accuracy in measuring body fat [22]. Dual-energy X-ray absorptiometry (DXA) can accurately determine the quantity and distribution of body fat with a low margin of error [22]. Moreover, many critical confounding factors potentially affecting both dietary Se intake and body composition have been poorly controlled. To date, no studies specifically designed to investigate the relationship between dietary Se intake and systematic measures of obesity have been completed.

Therefore, we designed the present study to investigate the relationship between dietary Se intake and a panel of body composition parameters, measured by DXA, in a large population-based study with systematic control of major confounding factors.

## 2. Subjects and Methods

### 2.1. Subjects

All participants were from the ongoing CODING (Complex Diseases in the Newfoundland population: Environment and Genetics) study [22,23,24,25,26]. Eligibility for the CODING study was based on the following inclusion criteria: (1) ≥19 years of age; (2) at least a third generation Newfoundlander; (3) without serious metabolic, cardiovascular, or endocrine diseases; and (4) not pregnant at the time of the study. Ethics approval was obtained from the Health Research Ethics Authority, Memorial University, St. John’s, NL, Canada, with Project Identification Code #10.33 (latest date of approval: 21 January 2015). All subjects provided written and informed consent before participation in this study. Detailed information regarding the CODING Study was reported in our previously published papers [22,23,24,25,26].

**Figure 1:**
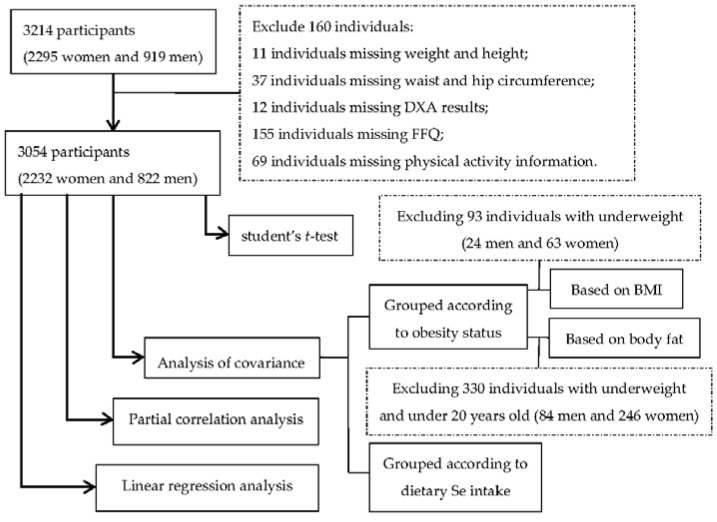
Flow-chart of subject selection for analyses.

A total of 3214 participants including 2295 women and 919 men were initially included. Among them 160 individuals were excluded due to incomplete or missing data, including weight and height for 11 individuals, waist and hip circumference for 37 individuals, DXA results for 12 individuals, food frequency questionnaire (FFQ) for 155 individuals, and physical activity information for 69 individuals (Figure 1).

### 2.2. Anthropometric Measurements

Anthropometrics were measured following a 12-h overnight fast. Trained personnel obtained anthropometric measurements for each subject using standard procedures. Standing height was measured using a fixed stadiometer (to the nearest 0.1 cm). After fully emptying their bladders, subjects wore standard hospital gowns for all weight measurements using a platform manual scale balance (Health O Meter, Bridgeview, IL, USA; nearest 0.1 kg). BMI (kg/m^2^) was calculated as weight in kilograms divided by height squared in meters. WC was measured at the midway point between the iliac crest and the lower rib, and hip by the maximum circumference over the buttocks below the iliac crest. Waist-hip ratio (WHR) was determined by dividing WC by hip circumference.

### 2.3. Body Composition Measurements

Body composition measurements, including total body fat percentage (BF%), trunk fat percentage (TF%), android fat percentage (AF%), and gynoid fat percentage (GF%), were taken in a supine position, utilizing DXA (Lunar Prodigy; GE Medical Systems, Madison, WI, USA) with the Lunar Prodigy software system, which has the capacity to distinguish each of these regions. The trunk fat region is measured from the top of the shoulders to the top of the iliac crest, the android fat region is represented by the distance from the top of the second lumbar vertebra to the top of the iliac crest, and the gynoid fat region extends down the iliac crest twice the height of the android area. The enCORE (Version 12.2, 2008, GE Medical Systems) software package was used for DXA data acquisition. Daily quality assurance was performed on the DXA scanner, and the typical coefficient of variation was 1.3% during the study period [23,24,25,26].

### 2.4. Dietary Assessment

Dietary intake for each participant was assessed by using a 124-item semi-quantitative Willett FFQ [27], which is one of the most commonly used dietary questionnaires for large scale epidemiological studies [28]. The Willett FFQ asks subjects to indicate the number of weekly servings of common food items consumed over the past 12 months. The FFQ was completed by each participant at the date of appointment. The NutriBase Clinical Nutrition Manager (version 8.2.0; CybersoftInc, Phoenix, AZ, USA) software package was used to convert weekly serving values into mean daily serving values to calculate the total daily intakes of calorie (kcal/day) and Se (μg/day) for each individual [23,25]. Dietary Se intake was expressed as per kilogram body weight (μg/kg/day).

### 2.5. Physical Activity Assessment and Other Information

Physical activity patterns were measured using the ARIC Baecke Questionnaire, which consists of a Work Index, Sports Index, and Leisure Time Activity Index [23,25]. In addition, all participants completed a self-administered screening questionnaire, which was used to collect information about their personal health history. Women completed an additional questionnaire regarding menstrual history and menopausal status (pre- or post-menopausal).

### 2.6. Data Analyses

All data are presented as mean ± standard error (SE). Calorie and dietary Se intake were log-transformed to normalize data distributions to perform effective statistical analysis. Anthropometrics, body composition, dietary intake and physical activity were compared between men and women using independent student’s *t*-test.

According to the criteria recommended by the World Health Organization [29], obesity status was categorized based on BMI, as normal weight (18.5–24.9 kg/m^2^), overweight (25.0–29.9 kg/m^2^), or obese (≥30 kg/m^2^). Obese subjects were further classified into three subgroup: obese class I (30.0–34.9 kg/m^2^), obese class II (35.0–39.9 kg/m^2^), and obese class III (≥40.0 kg/m^2^). The number of underweight subjects (BMI < 18.5kg/m^2^) was too small (*n* = 93, men/women = 24/69) to perform meaningful statistical analysis, so they were excluded from the analysis (Figure 1). Subjects were also divided into three groups based on body fat percentage according to age and gender specific criteria recommended by Bray [30]. Underweight subjects were excluded from the analysis due to their small numbers, and subjects under 20 years of age were also excluded due to lack of available criteria for this age group (Figure 1). Dietary Se intake was analyzed by analysis of variance and covariance controlling for age, total calorie intake and physical activity. Analyses on obesity measurements were performed when participants were divided into tertiles (low, medium, or high) based on dietary Se intake using analysis of variance and covariance controlling for age, total calorie intake and physical activity.

Partial correlation analysis controlling for age, total calorie intake, and physical activity was subsequently applied to determine the correlation between dietary Se intake and obesity measurements in both men and women, and also in obese groups separately. To overcome the possible influence of smoking, alcohol consumption, medication use, and menopausal status, participants were also divided into subgroups according to smoking status (yes or no), alcohol consumption (yes or no) and medication use (yes or no). Women were further divided into pre- or post-menopausal groups according to their menopausal status.

Finally, stepwise multiple linear regression analysis was used to evaluate the contribution of dietary Se intake to obesity among women or men. Weight, BMI, WC, WHR, TF%, AF%, GF%, and BF% were used as dependent variables and dietary Se intake, age, total calorie intake and physical activity were used as independent variables.

All statistical analyses were performed using SPSS 20.0 (SPSS Inc., Chicago, IL, USA). All tests were two-sided and a *p* < 0.05 was considered to be statistically significant.

## 3. Results

### 3.1. Body Composition and Dietary Se Intake

Physical and dietary characteristics of the entire cohort, as well as male and female subsets, are presented in Table 1. Women were on average 3 years older than men. Weight, BMI, WC, WHR and physical activity were significantly greater in men than women (*p* < 0.001). However, TF%, AF%, GF%, and BF% were significantly lower in men than in women (*p* < 0.001). Total calorie and Se intake were significantly higher in men than in women when differences in body weight were not adjusted for (*p* < 0.001). Mean dietary Se intake was 108.10 μg/day (125.42 μg/day in men and 101.78 μg/day in women), which is similar to the estimated dietary Se intake of the general Canadian population [31]. However, when body weight was adjusted for, (e.g., when dietary Se intake was expressed by μg/kg/day), there was no significant difference between men and women (*p* = 0.10).

**Table 1:** Obesity measurements and dietary Se intake according to gender.

	Entire Cohort (*n* = 3054)	Women (*n* = 2232)	Men (*n* = 822)	*p*
Age (year)	42.92 ± 0.24	43.71 ± 0.27	40.63 ± 0.49	<0.001
Weight (kg)	74.06 ± 0.29	69.50 ± 0.29	86.60 ± 0.54	<0.001
BMI (kg/m^2^)	26.70 ± 0.90	26.32 ± 0.11	27.71 ± 0.16	<0.001
WC (cm)	91.89 ± 0.25	89.85 ± 0.29	97.40 ± 0.46	<0.001
WHR	0.91 ± 0.01	0.89 ± 0.01	0.97 ± 0.02	<0.001
TF%	36.36 ± 0.18	38.62 ± 0.19	30.13 ± 0.34	<0.001
AF%	41.43 ± 0.21	43.43 ± 0.23	35.84 ± 0.41	<0.001
GF%	40.25 ± 0.18	44.56 ± 0.14	28.39 ± 0.28	<0.001
BF%	34.06 ± 0.17	37.34 ± 0.17	25.05 ± 0.28	<0.001
Calorie intake (kcal/d)	1976.36 ± 16.20	1867.03 ± 17.15	2281.53 ± 36.17	<0.001
Physical activity	8.28 ± 0.03	8.17 ± 0.03	8.52 ± 0.06	<0.001
Dietary Se intake (μg/day)	108.10 ± 1.04	101.78 ± 1.06	125.42 ± 2.52	<0.001
Dietary Se intake (μg/kg/day)	1.51 ± 0.02	1.51 ± 0.02	1.50 ± 0.03	0.10

All values are presented as means ± SEs. BMI, Body mass index; WC, Waist circumference; WHR, Waist-hip ratio; TF, trunk fat, which is the region from the top of the shoulders to the top of the iliac crest; AF, android fat, which is the region from the top of the second lumbar vertebra to the top of the iliac crest; GF, gynoid fat, which is the region extending down the iliac crest, and which is twice the height of the android area.

### 3.2. Variations in Dietary Se Intake Based on Obesity Status

When dietary Se intake was presented as μg/kg/day, after controlling for age, calorie intake and physical activity, a significantly lower dietary Se intake—from normal weight to overweight and obese groups—was revealed in both men and women (*p* < 0.001). Compared with normal weight subjects, dietary Se intake was 15% lower in overweight women, 31% lower in obese women, 15% lower in overweight men, and 28% lower in obese men (Table 2). As observed in the general population groups, when dietary Se intake was presented as μg/kg/day, a significant difference in dietary Se intake was revealed in obese groups. Compared to the obese class I group, dietary Se intake was 11% and 24% less in obese class II and III women, respectively. Similarly, male subjects in obese class II and III had dietary Se intake 12% and 21% lower than males categorized as obese class I (Table 2). The significance of these results was confirmed when obesity status was evaluated based on DXA-determined body fat percentage (Table 2).

**Table 2:** Dietary Se intake according to obesity status ^1^.

Grouped According to BMI ^2^		Normal Weight	Overweight	Obese	*p*-Trend
Women	*N*	1004	722	437	
	Dietary Se intake (μg/day)	100.76 ± 1.04	101.85 ± 1.21	104.08 ± 1.58	0.71
	Dietary Se intake (μg/kg/day)	1.70 ± 0.02	1.44 ± 0.02	1.18 ± 0.03	<0.001
	Dietary Se intake decline		−15% ^4^	−31% ^4^	
Men	*N*	234	350	214	
	Dietary Se intake (μg/day)	122.52 ± 2.98	125.37 ± 2.37	128.74 ± 3.07	0.54
	Dietary Se intake (μg/kg/day)	1.74 ± 0.04	1.48 ± 0.03	1.26 ± 0.04	<0.001
	Dietary Se intake decline		−15% ^4^	−28% ^4^	
**Grouped According to BMI ^2^**		**Obese Class I**	**Obese Class II**	**Obese Class III**	***p*-Trend**
Women	*N*	294	95	48	
	Dietary Se intake (μg/day)	102.49 ± 2.16	103.98 ± 3.79	104.18 ± 5.41	0.93
	Dietary Se intake (μg/kg/day)	1.22 ± 0.03	1.09 ± 0.04	0.93 ± 0.06	<0.001
	Dietary Se intake decline		−11% ^5^	−24% ^5^	
Men	*N*	158	45	11	
	Dietary Se intake (μg/day)	114.22 ± 3.02	116.35 ± 5.6	119.96 ± 11.81	0.29
	Dietary Se intake (μg/kg/day)	1.14 ± 0.03	1.00 ± 0.06	0.90 ± 0.12	0.001
	Dietary Se intake decline		−12% ^5^	−21% ^5^	
**Grouped According to Body Fat ^3^**		**Normal Weight**	**Overweight**	**Obese**	***p*-Trend**
Women	*N*	664	542	780	
	Dietary Se intake (μg/day)	99.47 ± 1.26	99.87 ± 1.36	100.28 ± 1.16	0.87
	Dietary Se intake (μg/kg/day)	1.68 ± 0.02	1.51 ± 0.02	1.25 ± 0.02	0.001
	Dietary Se intake decline		−10% ^4^	−26% ^4^	
Men	*N*	254	199	285	
	Dietary Se intake (μg/day)	123.96 ± 2.71	118.78 ± 2.96	120.74 ± 2.53	0.79
	Dietary Se intake (μg/kg/day)	1.64 ± 0.03	1.41 ± 0.04	1.25 ± 0.03	<0.001
	Dietary Se intake decline		−14% ^4^	−24% ^4^	

^1^ Data were assessed with Covariance controlling for age, total calorie intake, and physical activity. All values are presented as means ± SEs; ^2^ The following subdivision was grouped by BMI according to the criteria of the World Health Organization. Subjects who were underweight (*n* = 93, men/women = 24/69) were excluded from this analysis; ^3^ Subgroup were created by percent of body fat according to the age and gender specific criteria recommended by Bray. Subjects who were underweight or under 20 years of age (*n* = 330, men/women = 84/246) were excluded from this analysis; ^4^ Percent of dietary Se intake = ((dietary Se intake in normal weight group—dietary Se intake in overweight or obese group)/dietary Se intake in normal weight group) × 100%; ^5^ Percent of Se intake = ((dietary Se intake in obese class I group—dietary Se intake in obese class II or obese class III group)/dietary Se intake in Obese class I group) × 100%.

However, when dietary Se intake was expressed in μg/day, there was no significant difference in dietary Se intake among general population groups and obesity groups after controlling for age, calorie intake and physical activity (Table 2).

### 3.3. Variations in Obesity Measurements According to Amount of Dietary Se Intake

When subjects were grouped into tertiles (low, medium, or high) according to dietary Se intake (μg/kg/day), a significant inverse dose-dependent relationship was discovered between obesity severity, indexed by all body composition measurements (weight, BMI, WC, WHR, TF%, AF%, GF% and BF%), and dietary Se intake in both men and women after controlling for age, total calorie intake and physical activity (*p* < 0.01 for all, Table 3). Among all obesity measurements, changes in body weight and AF% in women and TF% and BF% in men were the most pronounced. As compared to the low Se intake group, body weight and AF% in the high Se intake group were 11% lower in women, while TF% and BF% in the high Se intake group in men were 16% lower, respectively.

For each 1 μg/kg/day increase in dietary Se intake, average weight, BMI, WC, and WHR decreased by 8.39 kg, 2.98 kg/m^2^, 8.03 cm, and 0.02 in women, and by 8.85 kg, 2.34 kg/m^2^, 7.39 cm, and 0.02 in men, respectively. Likewise, TF%, AF%, GF% and BF% were reduced by 4.58%, 5.56%, 3.05% and 4.16% in women, and by 5.43%, 5.94%, 4.19% and 4.45% in men, respectively (Table 3). Dietary Se intake (μg/kg/day) alone accounted for 9%–27% of the variations in body fat.

Similar results were also seen when dietary Se intake was expressed as μg/day. However, statistical significance was achieved only for the trend of rising body weight in women with decreasing dietary Se intake (*p* = 0.01), BMI, WC, WHR, TF%, AF%, GF%, and BF% did not differ significantly among the groups in both genders (Table 3).

**Table 3:** Variations in obesity measurements according to dietary Se intake.

Dietary Se Intake (μg/Day)		Low	Medium	High	*p*-Trend	
*n* (Women/Men)		744/274	744/274	744/274		
Women	Se (μg/day)	14.16~80.64	80.64~117.06	117.06~669.77		
	Weight (kg)	68.06 ± 0.59	69.51 ± 0.49	70.97 ± 0.59	0.01	
	BMI (kg/m^2^)	25.97 ± 0.22	26.31 ± 0.18	26.71 ± 0.22	0.10	
	WC (cm)	89.50 ± 0.57	89.75 ± 0.48	90.41 ± 0.57	0.57	
	WHR	0.89 ± 0.003	0.89 ± 0.002	0.89 ± 0.003	0.64	
	TF%	38.25 ± 0.36	38.64 ± 0.30	39.01 ± 0.36	0.43	
	AF%	43.17 ± 0.44	43.47 ± 0.37	43.75 ± 0.44	0.73	
	GF%	44.19 ± 0.28	44.83 ± 0.23	44.72 ± 0.28	0.23	
	BF%	36.90 ± 0.32	37.43 ± 0.26	37.72 ± 0.31	0.26	
Men	Se (μg/day)	20.13~92.07	92.07~133.59	133.59~683.93		
	Weight (kg)	84.19 ± 1.13	87.20 ± 0.90	88.27 ± 1.15	0.07	
	BMI (kg/m^2^)	27.24 ± 0.33	28.03 ± 0.26	27.83 ± 0.33	0.16	
	WC (cm)	96.95 ± 0.89	97.89 ± 0.71	97.26 ± 0.90	0.65	
	WHR	0.97 ± 0.004	0.98 ± 0.003	0.98 ± 0.004	0.40	
	TF%	30.44 ± 0.63	30.78 ± 0.50	29.14 ± 0.65	0.14	
	AF%	36.53 ± 0.77	36.39 ± 0.61	34.60 ± 0.78	0.18	
	GF%	28.73 ± 0.56	28.83 ± 0.44	27.62 ± 0.57	0.25	
	BF%	25.30 ± 0.54	25.63 ± 0.43	24.20 ± 0.55	0.12	
**Dietary Se Intake (μg/kg/Day)**		**Low**	**Medium**	**High**	**Variations ^1^**	***p*-Trend**
***n* (Women/Men)**		**744/274**	**744/274**	**744/274**		
Women	Se (μg/kg/day)	0.14~1.12	1.12~1.66	1.66~13.46		
	Weight (kg)	78.76 ± 0.52	69.03 ± 0.44	60.70 ± 0.52	−8.93	<0.001
	BMI (kg/m^2^)	29.52 ± 0.19	26.11 ± 0.16	23.35 ± 0.19	−2.98	<0.001
	WC (cm)	97.99 ± 0.52	89.12 ± 0.44	82.55 ± 0.51	−8.03	<0.001
	WHR	0.91 ± 0.003	0.89 ± 0.002	0.88 ± 0.003	−0.013	<0.001
	TF%	43.09 ± 0.33	38.48 ± 0.28	34.35 ± 0.33	−4.58	<0.001
	AF%	48.65 ± 0.41	43.40 ± 0.35	38.34 ± 0.40	−5.56	<0.001
	GF%	47.02 ± 0.27	44.76 ± 0.23	41.95 ± 0.26	−3.05	<0.001
	BF%	41.26 ± 0.29	37.25 ± 0.25	33.55 ± 0.29	−4.16	<0.001
Men	Se (μg/kg/day)	0.22~1.04	1.05~1.60	1.61~9.59		
	Weight (kg)	95.56 ± 1.04	87.42 ± 0.84	76.58 ± 1.07	−8.85	<0.001
	BMI (kg/m^2^)	30.24 ± 0.30	27.88 ± 0.25	24.96 ± 0.31	−2.34	<0.001
	WC (cm)	104.71 ± 0.81	97.75 ± 0.66	89.61 ± 0.84	−7.39	<0.001
	WHR	0.99 ± 0.004	0.98 ± 0.003	0.96 ± 0.004	−0.020	<0.001
	TF%	34.15 ± 0.59	31.45 ± 0.48	24.70 ± 0.61	−5.43	<0.001
	AF%	40.60 ± 0.72	37.13 ± 0.58	29.72 ± 0.72	−5.94	<0.001
	GF%	31.54 ± 0.53	29.10 ± 0.43	24.47 ± 0.54	−4.19	<0.001
	BF%	28.61 ± 0.50	25.93 ± 0.40	20.53 ± 0.52	−4.45	<0.001

Data were assessed with Covariance controlling for age, total calorie intake, and physical activity. All values are presented as mean ± SE. BMI, Body mass index; WC, Waist circumference; WHR, Waist-hip ratio; TF, trunk fat, which is the region from the top of the shoulders to the top of the iliac crest; AF, android fat, which is the region from the top of the second lumbar vertebra to the top of the iliac crest; GF, gynoid fat, which is the region extending down the iliac crest twice the height of the android area. ^1^ Variations in obesity measurements with higher dietary Se intake in μg/kg/day.

### 3.4. Correlation between Dietary Se Intake and Obesity Measurements

The correlations between dietary Se intake and measures of obesity are presented in Table 4. In both men and women, dietary Se intake was negatively associated with the majority of obesity measurements (*p* < 0.01 for all), except for body weight in men and women, and BMI and WHR in women (*p* > 0.05). After adjusting for age, total calorie intake and physical activity, the negative correlation between dietary Se intake (μg/kg/day) and TF% (*r*’ = −0.43 for men and −0.41 for women, *p* < 0.001 for both), AF% (*r*’ = −0.41 for men and −0.40 for women, *p* < 0.001 for both), GF% (*r*’ = −0.35 for men and −0.30 for women, *p* < 0.001 for both) and BF% (*r*’ = −0.43 for men and −0.41 for women, *p* < 0.001 for both) were statistically significant. These significant negative correlations hold in obese men and women, after controlling for confounding factors.

**Table 4:** Correlation between dietary Se intake and obesity measurements.

		All Women (*n* = 2232)	Obese Women (*n* = 437)	All Men (*n* = 822)	Obese Men (*n* = 214)
		*r* (*p*)	*r*’ (*p*)	*r* (*p*)	*r*’ (*p*)
Dietary Se intake (μg/day)	Weight	0.02 (0.32)	0.005 (0.92)	0.08 (0.02)	0.12 (0.08)
	BMI1	0.01 (0.53)	0.004 (0.93)	0.06 (0.09)	0.07 (0.32)
	WC1	−0.03 (0.13)	−0.120 (0.01)	0.01 (0.76)	0.03 (0.68)
	WHR	−0.03 (0.15)	−0.129 (0.01)	0.04 (0.28)	0.04 (0.57)
	TF%	−0.01 (0.66)	−0.046 (0.34)	−0.07 (0.07)	0.001 (0.99)
	AF%	−0.02 (0.31)	−0.031 (0.51)	−0.08 (0.03)	−0.06 (0.41)
	GF%	−0.004 (0.84)	0.012 (0.80)	−0.07 (0.05)	−0.10 (0.14)
	BF%	−0.001 (0.95)	−0.024 (0.62)	−0.06 (0.09)	0.01 (0.91)
Dietary Se intake (μg/kg/day)	Weight	−0.52 (<0.001)	−0.37 (<0.001)	−0.46 (<0.001)	−0.30 (<0.001)
	BMI	−0.49 (<0.001)	−0.29 (<0.001)	−0.43 (<0.001)	−0.25 (<0.001)
	WC	−0.49 (<0.001)	−0.36 (<0.001)	−0.46 (<0.001)	−0.29 (<0.001)
	WHR	−0.19 (<0.001)	−0.13 (0.01)	−0.22 (<0.001)	−0.05 (0.51)
	TF%	−0.41 (<0.001)	−0.12 (0.01)	−0.43 (<0.001)	−0.12 (0.08)
	AF%	−0.40 (<0.001)	−0.12 (0.01)	−0.41 (<0.001)	−0.19 (0.01)
	GF%	−0.30 (<0.001)	−0.05 (0.26)	−0.35 (<0.001)	−0.21 (0.002)
	BF%	−0.41 (<0.001)	−0.16 (0.001)	−0.43 (<0.001)	−0.16 (0.02)

Partial correlations between dietary Se intake and obesity measurements controlled for age, total calorie intake, and physical activity. BMI, Body mass index; WC, Waist circumference; WHR, Waist-hip ratio; TF, trunk fat, which is the region from the top of the shoulders to the top of the iliac crest; AF, android fat, which is the region from the top of the second lumbar vertebra to the top of the iliac crest; GF, gynoid fat, which is the region extending down the iliac crest twice the height of the android area. *r*’, partial correlation coefficient.

To further investigate the influence of additional covariates, participants were subdivided based on smoking, alcohol consumption, medication use and menopausal status (Table 5). For each subset, partial correlation analyses were conducted controlling for confounding factors: age, physical activity and total caloric intake. Dietary Se intake remained negatively correlated with TF%, AF%, GF% and BF% in these subgroups without smoking, alcohol, and medication, while as same as in the pre-menopausal and post-menopausal subgroup.

Findings from multiple linear regression analysis are presented in Table 6. Dietary Se intake (μg/kg/day) was significantly associated with all obesity indexes including weight, BMI, WC, WHR, TF%, AF%, GF% and BF%. However, dietary Se intake (μg/day) was only associated with TF%, AF%, GF% and BF% in men, not in women.

**Table 5:** Partial correlations between dietary Se intake (μg/kg/day) and body compositions based on smoking, alcohol consumption, medication use and menopausal status.

	Smoking		Alcohol		Medication		Menopausal	
	No *r*’	Yes *r*’	No *r*’	Yes *r*’	No *r*’	Yes *r*’	Pre *r*’	Post *r*’
**Women**								
Weight	−0.03	−0.03	−0.04	−0.02	−0.03	−0.03	−0.05	0.02
BMI	−0.04	−0.01	−0.04	−0.04	−0.04	−0.03	−0.05	0.01
WC	−0.07 **	−0.19 **	−0.09 **	−0.06	−0.08	−0.08	−0.10	0.04
WHR	−0.03	−0.16 *	−0.05 *	−0.04	−0.03	−0.05	−0.07	−0.02
TF%	−0.05 *	−0.05	−0.06 *	−0.02	−0.07 *	−0.04	−0.07 *	−0.01 *
AF%	−0.06 *	−0.10	−0.07 **	−0.04	−0.09 *	−0.05	−0.08 *	−0.02 *
GF%	−0.04 *	−0.04	−0.05 *	−0.01	−0.07 *	−0.01	−0.06 *	0.03 *
BF%	−0.05 *	−0.02	−0.06 *	−0.01	−0.06*	−0.03	−0.07 *	0.01 *
**Men**								
Weight	0.06	0.02	−0.03	0.19	0.02	0.13		
BMI	0.04	−0.03	−0.01	0.17	0.01	0.08		
WC	−0.01	−0.10	−0.04	0.11	−0.04	0.03		
WHR	0.04	−0.07	−0.01	0.14	0.06	−0.03		
TF%	−0.12 **	−0.01	−0.123 **	0.03	−0.13 **	−0.04		
AF%	−0.12 **	−0.08	−0.126 **	−0.02	−0.13 **	−0.07		
GF%	−0.10 **	−0.09	−0.104 **	−0.06	−0.13 **	−0.03		
BF%	−0.11 **	−0.04	−0.117 **	−0.03	−0.14 **	−0.01		

Controlling for age, total calorie intake, and physical activity. BMI, Body mass index; WC, Waist circumference; WHR, Waist-hip ratio; TF, trunk fat, which is the region from the top of the shoulders to the top of the iliac crest; AF, android fat, which is the region from the top of the second lumbar vertebra to the top of the iliac crest; GF, gynoid fat, which is the region extending down the iliac crest twice the height of the android area. *r*’: partial correlation coefficient; *, *p* < 0.01; ** *p* < 0.01.

**Table 6:** Regression analysis of dietary Se intake with obesity related indexes.

	Women (*n* = 2232)			Men (*n* = 822)		
	*R*^2^	β	*p* Value	*R*^2^	β	*p* Value
Dietary Se intake (μg/day)						
weight	0.051	0.032	0.348	0.044	0.095	0.139
BMI	0.085	0.026	0.447	0.090	0.049	0.430
WC	0.136	−0.052	0.117	0.199	0.010	0.862
WHR	0.039	−0.049	0.157	0.100	0.063	0.310
TF%	0.176	−0.013	0.692	0.260	−0.128	0.023
AF%	0.153	−0.030	0.352	0.249	−0.149	0.009
GF%	0.106	−0.009	0.786	0.122	−0.142	0.021
BF%	0.175	0.001	0.985	0.221	−0.121	0.036
Dietary Se intake (μg/kg/day)						
Weight	0.315	−0.781	<0.001	0.266	−0.821	<0.001
BMI	0.301	−0.708	<0.001	0.283	−0.761	<0.001
WC	0.341	−0.692	<0.001	0.374	−0.734	<0.001
WHR	0.074	−0.289	<0.001	0.144	−0.368	<0.001
TF%	0.315	−0.569	<0.001	0.405	−0.670	<0.001
AF%	0.286	−0.557	<0.001	0.380	−0.642	<0.001
GF%	0.191	−0.445	<0.001	0.229	−0.580	<0.001
BF%	0.315	−0.571	<0.001	0.373	−0.682	<0.001

## 4. Discussion

To the best of our knowledge, this is the first study specifically designed to examine the association between dietary Se intake and a full panel of obesity measurements with systematic control of major confounding factors in a large adult population. The most significant finding in the present study was that obesity and degree of obesity were associated with low dietary Se intake in the general adult population. Every 1 μg/kg/day increase in dietary Se intake corresponded to a 3%–6% decrease in body fat percentage.

To date, there is only one reported cross-sectional study evaluating the relationship between dietary Se intake and obesity in school children aged 8–13 years old [32]. In that study, children with BMI >85th percentile had significantly lower dietary Se intake (μg/kg/day) than normal weight children, after adjusting for energy intake. The remaining studies evaluated the association with other biological samples rather than dietary Se intake. Data were extrapolated from nutrition surveys or studies designed to examine the relationship between Se and cardiovascular disease, diabetes or cancer. For example, data from the 1999 to 2004 US National Health and Nutrition Examination Survey (NHANES) showed that children at high risk of overweight were also at greater risk of dietary Se deficiency [33]. In a survey from the NHANES 2003–2004, subjects were divided into quartiles based on serum selenium, and BMI decreased and dietary Se intake (μg/day) increased with increasing serum Se levels [34]. A positive correlation between serum Se concentration and dietary Se intake (μg/day) was found, and a negative correlation between serum Se and BMI was reported [32,35]. Similar findings were revealed in a cancer study in Northern Italy [36]. Additionally, an adverse association between anthropometric measurements and serum/plasma Se levels has been reported [19,20,21,37,38,39].

A variety of factors may affect body composition and dietary Se intake, including age, total dietary calorie intake and physical activity. Body composition, food choice and intake may change with age, making this an important confounding factor to be controlled for in analysis [40]. Higher dietary calorie intake is a central risk factor for obesity, and is significantly correlated with dietary Se intake as well [41]. This was observed in the present study (*r* 0.13 to 0.27, not shown). Physical activity is likely one of the most important variables determining the amount of body fat [42]. In addition, there are notable gender differences in the amount of body fat and fat distribution. On average, women have 12% more body fat than men in the Newfoundland population [26]. Difference in food consumption also exists between men and women [43]. Therefore, separate analyses were performed for men and women to eliminate the effect of gender on our results. In women, menopause results in profound hormonal changes, which may predispose to increased adiposity—a factor we also considered in our study [44]. We found a similar correlation between dietary Se intake and body composition in both premenopausal and postmenopausal women. Smoking, alcohol consumption and medication use are potentially important covariates as well, since they may affect appetite and body weight regulation [45,46]. After separating the subjects according to these covariates, the association between dietary Se intake and body composition remained significant. It should be noted that the systematic control of major confounding factors in this study enabled us obtaining accurate and reliable findings.

The effect of dietary Se intake on body fat is supported by data from animal interventional experiments. Wang *et al*. [47] found that body weight significantly decreased and the ratio of adipose to body weight dropped when rats were treated with high doses of Se (200 μg/kg/day). This was due to enhanced lipolysis in adipose tissue and hepatic accumulation free fatty acids. Netto *et al.* [48] obtained a similar result. However, two small interventional studies in healthy human volunteers revealed contradictory findings [49,50]. In the study carried out by Hawkes *et al.* [49], body weight increased in the high Se group (297 μg/day) while weight decreased in the low Se group (14 μg/day) after 64 days of treatment, although energy intake was the same in both groups (5 *vs.* 6 in each group). In the study conducted by Navas-Carretero *et al.* [50], consumption of Se-enriched chicken (11 subjects) did not result in more weight loss than consumption of Se-non-enriched chicken (13 subjects). However caution must be exercised in interpreting these results, as the very small sample size may have contributed to this discrepancy.

The second major finding in our study is that the beneficial association between dietary Se intake and body fat is not only significant in normal and overweight subjects but also in obese individuals. This finding supports the theory that appropriate dietary Se supplementation may be useful in the battle against obesity. Se supplementation would be a simple and cost-efficient intervention for both overweight and obese individuals. It should be emphasized, however, that the low dietary Se intake in obese individuals may be a consequence of long-term consumption of high-fat and high-sugar foods, as well as more sugar-sweetened beverages [51]. Such foods are typically low in Se content, and are negatively correlated with serum Se [52].

At present, the mechanisms underlying this beneficial effect of dietary Se on body fat remain largely unclear. However, evidence linking Se with adipogenesis does exist. Some earlier studies have utilized Se in the differentiation of primary pig and rat preadipocytes, as well as chicken embryonic fibroblasts, suggesting that Se may have proadipogenic potential [53,54]. However, a recent study showed that Se also inhibits adipogenesis through reduction of mRNA expression of peroxisome proliferator-activated receptor-γ and fatty acid synthase, while activating expression of transforming growth factor-β [18]. Furthermore, intra-peritoneal injections of sodium selenite reduce abdominal fat accumulation and adipocyte size in OLETF rats, supporting the anti-adipogenic role of Se *in vivo* [17].

In our study, dietary Se intake was expressed as μg/kg/day in all analyses. With substantial variations in body weight in the general human population, individuals of different body weight have different Se requirements. Therefore, one concern when Se is expressed as μg/day and large variations in body weight are not adjusted for in analysis is that results will be confounded. Consequently we included weight-based selenium intake (μg/kg/day) to eliminate this potential confounder. Furthermore, DXA measurement of body fat is more accurate than BMI and other field methods and best represents body adipose tissue with a low margin of error [22]. Our use of DXA in the entire study contributed to the reliability of our findings.

Nevertheless, several possible limitations exist in the presented study because it is a population-based association study. The cross-sectional study design does not allow the determination of cause and effect. Secondly, the current version of DXA analysis software does not specifically measure visceral fat, and so any possible association between dietary Se intake and visceral fat could not be determined. Therefore, further research investigating the association between dietary Se and central adiposity is required. Dietary Se is not the only marker of Se nutritional status; serum Se, GPx activity, and selenoprotein *p* are all important considerations. Further studies are needed to more closely evaluate their potential role—either alone or as a functional group if all measurements are available [55,56]. Finally, the FFQ is designed to assess habitual dietary intake by determining the frequency with which specific food items are consumed over a reference period. It is the most widely used dietary assessment method in large-scale epidemiological studies on macro- and micro-nutrient intakes. Moreover, the FFQ has been used to estimate dietary Se intake in previous studies [57,58,59,60]. However, it may not be as accurate for quantification of micronutrient intake, as compared with macronutrient intake [61].

## 5. Conclusions

In this large Newfoundland-population-based study, we were able to demonstrate that dietary Se intake has a strong inverse association with obesity and obesity severity in the general healthy, overweight and obese adult populations, independent of age, sex, total calorie intake and physical activity levels.
